# Epidemiological trends in mortality, event rates and case fatality of acute myocardial infarction from 2004 to 2015: results from the KORA MI registry

**DOI:** 10.1080/07853890.2021.2002926

**Published:** 2021-11-15

**Authors:** Christina Krämer, Christa Meisinger, Inge Kirchberger, Margit Heier, Bernhard Kuch, Christian Thilo, Jakob Linseisen, Ute Amann

**Affiliations:** a Independent Research Group Clinical Epidemiology, Helmholtz Zentrum München, German Research Center for Environmental Health (GmbH), Neuherberg, Germany; b Institute for Medical Information Processing, Biometry, and Epidemiology, Ludwig-Maximilians-Universität München, Munich, Germany; c Department of Internal Medicine I – Gastroenterology, Oncology and Endocrinology, Hospital of Friedrichshafen, Friedrichshafen, Germany; d Chair of Epidemiology, University of Augsburg at University Hospital Augsburg, Augsburg, Germany; e KORA Study Centre, University Hospital of Augsburg, Augsburg, Germany; f Institute of Epidemiology, Helmholtz Zentrum München, German Research Center for Environmental Health, Neuherberg, Germany; g Department of Internal Medicine I – Cardiology, University Hospital of Augsburg, Augsburg, Germany; h Department of Internal Medicine – Cardiology, Hospital of Nördlingen, Nördlingen, Germany; i Department of Medical Clinic I – Cardiology, Hospital of Rosenheim, Rosenheim, Germany

**Keywords:** Myocardial infarction, mortality, incidence, recurrence, case fatality, time trends, epidemiology, Germany

## Abstract

**Aim:**

This study examines epidemiological trends of acute myocardial infarction (AMI) in Germany from 2004–2015 across different age groups, using data of the population-based KORA myocardial infarction registry.

**Methods:**

Annual age-standardised, age-group- and sex-specific mortality and event rates (incident and recurrent) per 100,000 population as well as 28-day case fatality were calculated from all registered cases of AMI and coronary heart disease deaths in 25–74-year-olds from 2004–2015 and 75–84-year-olds from 2009–2015. Average annual percentage changes (AAPC) were calculated by joinpoint regression.

**Results:**

Mortality rates declined considerably among the elderly (75–84 years), in men by –6.0% annually, due to declines of case fatality by –3.0% and incidence rate by 3.4% and in women by –10.0%, driven by declines in incidence (–9.1%) and recurrence rate (–4.9%). Significant mortality declines also occurred in males, 65–74 years of age (AAPC –3.8%). Among the age groups 25–54 years and 55–64 years, there was no substantial decline in mortality, event rates or case fatality except for a decline of incidence rate in 55–64-year-old men (AAPC –1.8%).

**Conclusion:**

Inhomogeneous AMI trends across age-groups indicate progress in prevention and treatment for the population >64 years, while among <55-year-olds, we found no significant trend in AMI morbidity and mortality.

**KEY MESSAGES**
- Age standardised AMI mortality continued to decline from 2009 to 2015 in the study region.
- Declines in AMI mortality were driven by declines in event rates (both incidence and recurrence rates) and case fatality.
- AMI trends were inconsistent across different age groups with the strongest declines in mortality and event rates among the elderly population (75–84 years of age).

## Introduction

Coronary heart disease (CHD) is still one of the leading causes of death in most high-income countries within and outside Europe, although mortality rates have already decreased substantially since the early 1980s [1,2]. This decline in CHD mortality has largely been attributed to primary prevention measures like improved treatment and modification of cardiovascular risk factors such as arterial hypertension, hyperlipidaemia, diabetes mellitus and tobacco smoking, as well as to improvements in diagnostics and medical treatment of CHD, including secondary prevention [2,3].

The World Health Organisation (WHO) MONICA (Monitoring Trends and Determinants in Cardiovascular Disease) project contributed significantly to a global monitoring of CHD mortality trends. Due to its population-based registry setting it was possible to monitor both trends in case fatality and in acute myocardial infarction (AMI) event rates. Declining event rates had been identified as the major contributor to mortality declines in several populations up to 64 years in the 1980s [4].

In Germany the population-based MONICA/KORA (Cooperative Health Research in the Region of Augsburg) myocardial infarction registry showed a decline of AMI mortality from 1984 to 2002 among the population aged 25–74 years, which has mainly been explained by declines in recurrent events and 28-day case fatality [4,5]. We are now able to present trends up to the year 2015, including the 75- to 84-year-old population, which was added to the registry since 2009 in order to consider the fact that the elderly population constitute an increasing proportion of AMI patients. While most population-based studies including the population >74 years were focussed on trends in mortality rates [2,6–9] or incidence rates [8,10–12], only few have also investigated trends in recurrence rate or case fatality [13,14].

When looking at the young population (<55 years), an alarming slowdown of the mortality decline has been reported in the most recent years from several high-income countries [3,9,13]. Incidence rates, being a major contributor to mortality rates, have even begun to rise among young people in Australia and Denmark within recent years [12,15]. Unfavourable trends in several cardiovascular risk factors, especially an increasing prevalence of obesity, are suspected to have contributed largely to these latest developments [2,3].

Continuous monitoring of CHD trends is crucial for the evaluation of preventive strategies [11] and the effectiveness of medical and invasive treatment measures [1,4]. Since age-standardised rates may mask opposing trends at different ages, an age-specific approach is required [16].

The objective of this study was to evaluate trends in AMI mortality, and event rates (of incident and recurrent events) as well as 28-day case fatality by sex and age-groups in a population-based registry setting for the population aged 25–74 years from 2004 to 2015 and also for the population aged 75–84 years from 2009 to 2015.

## Methods

### Data source

We used data from the population-based myocardial infarction (MI) registry in Augsburg, Germany, which was implemented as part of the WHO MONICA project in 1984 [5]. Since 1996 the registry has been continued as part of the KORA research program.

All cases of AMI and coronary deaths (hereinafter referred to as “events”) occurring in the 25- to 74-year-old inhabitants of the city of Augsburg and two surrounding counties (overall around 680,000 inhabitants) have been continuously registered since 1984 [5]. In 2009, the registry has been extended to the population up to 84 years.

Methods of case-finding, diagnostic classification of events, and data quality control have been described in detail in previous studies [5,17,18]. Briefly, all events were registered according to the WHO MONICA protocol [17,19]. Since 1 January 2001, AMI was defined according to the diagnostic criteria from the European Society of Cardiology and the American College of Cardiology [20].

Data collection of the KORA MI registry has been approved by the ethics committee of the Bavarian Medical Association (Bayerische Landesärztekammer) and AMI patients who actively participated in the registry gave written informed consent.

### Data collection and definitions

The procedure of data collection in the KORA MI registry depends on the survival status of the respective patient. Patients surviving at least 24 h after hospitalisation who gave their informed consent, were interviewed by trained nurses shortly after intensive care using a standardised questionnaire. A concluding review of the medical record and the discharge report were added to supplement the self-reported information. Like that, sociodemographic data (age, sex), data on medical and cardiovascular history (e.g. previous AMI or stroke), on the presence of cardiovascular risk factors, and on vital signs were collected. Information about the survival status within 28 days after the acute event was given through the tracing of vital status during hospitalisation and screening of death certificates, obtained from local health departments in the study region.

From the hospitalised AMI patients who refused to participate in further data collection, only sex, age, and date of the event were registered (*n* = 120 from 2004 to 2015). Those events are necessary for complete assessment of population-based rates and were included in this study in the calculation of the AMI event rate.

In cases of early coronary death (occurring before admission to hospital or within 24 h of hospitalisation), data gathering was performed anonymously by screening of death certificates in addition to a written questionnaire, routinely sent by the local health departments to the last treating physician. Due to the complexity of this anonymous procedure, the number of missing information on cardiovascular history and cardiovascular risk factors was higher in the sub-group of early death events compared to those surviving longer.

An event was considered as first (incident) event if the patient’s history was free from a previous clinically recognised AMI, otherwise the event was considered as recurrent. An event in the same patient was considered a new event, when it occurred later than 28 days after any preceding AMI event. Patient characteristics in cardiovascular risk factors were counted as “yes” if either the information on the available documents (death certificate or medical record) or the patient’s answer in the interview reported a medically confirmed diabetes mellitus, arterial hypertension, hyperlipidaemia or history of stroke. Patients were categorised as current smokers when indicating smoking at the time of the event in the patient interview.

### Study population

In the current analyses, we included all residents of the Augsburg study region that had been recorded with AMI or coronary death in the KORA MI registry aged 25–74 years between 2004 and 2015 and aged 75–84 years between 2009 and 2015. No further exclusion criteria has been applied.

### Statistical analysis

All analyses were performed separately for women and men and for each calendar year from 2004 to 2015. Frequency of cardiovascular risk factors and age-specific rates were calculated within the following age-groups: 25–54, 55–64, 65–74, and 75–84 years. For descriptive purpose on cardiovascular risk factors, men and women within the four age-groups were compared using the chi-square test. The number of inhabitants in the study region in each age group and year stem from population data published by the German Federal Statistical Office [21].

Event rates were calculated by dividing the total number of AMI and coronary deaths by the mid-year population of the respective calendar year in the corresponding age-group and were expressed per 100,000 inhabitants. Incidence rates were calculated similarly, using the number of first events in the numerator. Recurrence rates were calculated using the number of recurrent events in the numerator. For the calculation of mortality rates, we used the total number of deaths within 28 days following the acute event in the numerator. 28-day case fatality (in %) was calculated respectively as the proportion of deaths to all events multiplied by 100.

To describe the sex-specific overall trend, mortality rate, event rate, incidence rate, and recurrence rate were directly age standardised within 10-year age-groups to the German population of 2015 [21] ages 25–74 years for the time period 2004–2015 and ages 25–84 years for the time period 2009–2015. Similar to the method used by Löwel et al. [5], case-fatality rates were age-standardised using the age-specific weights of all registered events within the respective time period.

To evaluate trends in the age-standardised and age-specific population rates, joinpoint regression, using the software Joinpoint, Version 4.7.0.0 (Statistical Research and Applications Branch, National Cancer Institute, Bethesda, Maryland) [22], was applied. This method detects years in which significant changes in trends appeared (so called “joinpoints”), by using a grid search method and estimates annual percentage changes (APC) in rates between these joinpoints and average annual percentage change (AAPC) together with 95% confidence intervals (CI) over the whole investigated time period from 2004 to 2015 and 2009 to 2015, respectively. In our analysis we focussed on AAPC over the whole observed period because analysis of a shorter time period is prone to bias due to low absolute numbers, especially in young women.

The maximum number of joinpoints was set at two. Weighted Bayesian Information Criterion was used to determine the model that best fitted the data with the smallest number of joinpoints. The two-sided significance level was set at (*p* < 0.05) for all tests. To analyse constant percentage (rather than absolute) changes in the rates over time, we used a log-linear model.

Further statistical analyses were performed using SAS version 9.5 (SAS Institute Inc., Cary, North Carolina).

## Results

The numbers of all registered AMI and coronary deaths between 2004 and 2015 (*n* = 17,417), of first and recurrent events and survival after the events as well as the distribution of cardiovascular risk factors in all events are shown in Table 1 by age group and sex. About a third (31.0%, *n* = 5,390) of all registered events between 2004 and 2015 occurred in women. The proportion of events occurring in women was 45.1% when considering the oldest age group (75–84 years) from 2009 to 2015 (*n* = 2,288).

**Table 1.** Overview of all registered events (*n* = 17,417) in the KORA MI registry between 2004 and 2015 stratified by sex and age group.

		Men *(n* = 12,027; 69.1 %)				Women (*n* = 5,390; 31.0 %)			
		2004–2015			2009–2015^a^	2004–2015			2009–2015^a^
		AG 1	AG 2	AG 3	AG 4	AG 1	AG 2	AG 3	AG 4
		25–54 years	55–64 years	65–74 years	75–84 years	25–54 years	55–64 years	65–74 years	75–84 years
		*n* = 2,157	*n* = 2,727	*n* = 4,356	*n* = 2,787	*n* = 469	*n* = 722	*n* = 1,911	*n* = 2,288
Acute cardiac event, *n* (%) ^b^									
First event		1,667 (84.9)	1,870 (76.4)	2,691 (69.3)	1,479 (61.2)	388 (91.7)	541 (83.2)	1,295 (76.7)	1,474 (75.7)
Recurrent event		297 (15.1)	577 (23.6)	1,195 (30.8)	938 (38.8)	35 (8.3)	109 (16.8)	393 (23.3)	474 (24.3)
Survival, *n* (%) ^c^									
Died prehospital		402 (18.7)	614 (22.7)	1,147 (26.5)	982 (35.5)	96 (20.5)	160 (22.4)	553 (29.2)	927 (40.9)
Died within 24 hours		84 (3.9)	189 (7.0)	591 (13.6)	545 (19.7)	23 (4.9)	51 (7.2)	216 (11.4)	393 (17.3)
Died within 28 days		37 (1.7)	94 (3.5)	219 (5.1)	159 (5.7)	13 (2.8)	34 (4.8)	81 (4.3)	108 (4.8)
Survived > 28 days		1,622 (75.6)	1,811 (66.9)	2,376 (54.8)	1,083 (39.1)	336 (71.8)	468 (65.6)	1,045 (55.1)	838 (37.0)
Cardiovascular risk profile, n (%)									
Diabetes Mellitus^d^	Yes	428 (21.6)	869 (34.8)	1,633 (41.4)	1,039 (42.2)	102 (23.3)	251 (38.2)	739 (42.8)	864 (42.9)
Arterial Hypertension^e^	Yes	1,262 (63.4)	1,949 (77.7)	3,253 (82.3)	2,145 (86.7)	268 (61.6)	521 (78.6)	1,488 (85.1)**	1,804 (88.3)
History of stroke^f^	Yes	60 (3.1)	208 (8.6)	535 (14.3)	515 (21.7)	16 (3.8)	42 (6.7)	199 (12.1)*	431 (22.2)
Hyperlipidaemia^g^	Yes	1,114 (56.4)	1,473 (59.4)	2,261 (58.4)	1,279 (53.1)	203 (47.3)***	376 (57.6)	1,018 (60.1)	985 (50.0)*
Smoking^h^	Yes^i^	1,056 (66.1)	747 (42.1)	465 (20.2)	106 (10.6)	214 (64.8)	208 (45.6)	179 (19.0)	43 (5.9)***

AG: age group; CHD: Coronary Heart Disease.

^a^Registration in the population aged 75–84 years started in January 2009.

^b^Data on history of cardiac event available for *n* = 15,423 (10,714 male, 4,709 female) due to different sources of information for data collection depending on length of survival after the acute event (number of missing information on cardiovascular history was higher in the collective of prehospital deaths (*n* = 1,336) and died within 24 h (535) than in those surviving longer (*n* = 3)).

^c^Information not available for a total of 120 persons (72 men and 48 women) who refused to consent in further data collection.

^d^Data available for *n* = 15,723 (10,889 male, 4,834 female).

^e^Data available for *n* = 15,816 (10,928 male, 4,888 female).

^f^Data available for *n* = 15,113 (10,479 male, 4,634 female).

^g^Data available for *n* = 15,484 (10,737 male, 4,747 female).

^h^Data available for *n* = 9,127 (6,672 male, 2,455 female). Data refer only to patients surviving longer than 24 h after hospitalisation because information on smoking status was collected by patient interview during hospital stay.

^i^Smoking one or more cigarettes per day by the time of the event.

***p < .001; **p < .01; *p < .05; *p*-value of chi-square test for men versus women regarding cardiovascular risk factors.

### Cardiovascular risk factors

The proportion of all observed cardiovascular risk factors were similar in men compared to women within the respective age groups, with only a few exceptions (Table 1). In the age-group of 25–54 years, hyperlipidaemia was 56.4% among men and 47.3% in women. Among 65- to 74-year-olds, arterial hypertension was more frequent in women while history of a former stroke was more frequent among the male population. Current smoking was almost twice as high in 75- to 84-year-old men compared to women of the same age.

### Trends in mortality

**Figure 1.**
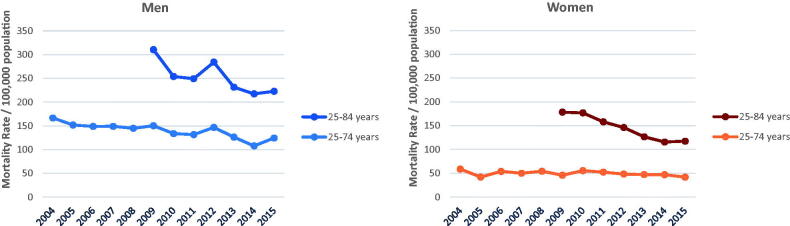
Age-standardised mortality rates of acute myocardial infarction in Augsburg study region, Germany, for men and women aged 25–74 years from 2004 to 2015 and for the population up to 84 years from 2009 to 2015.

**Figure 2.**
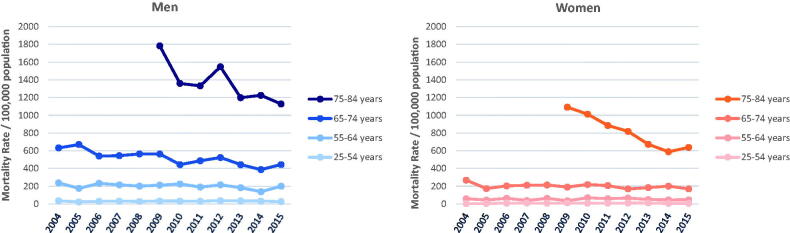
Age group- and sex-specific mortality rates of acute myocardial infarction in Augsburg study region, Germany, from 2004 to 2015.

As shown in Figure 1, age-standardised AMI mortality rates were about three times higher in men compared to women in the population aged 25–74 years and about twice as high in the population aged 25–84 years. In the oldest age group (75–84 years), mortality rates were several times higher in contrast to all younger age groups in both men (2015: 1,128 versus 445, 202 and 26 per 100,000) and even more pronounced in women (2015: 638 versus 169, 47 and 7 per 100,000) (see Figure 2).

Over the observed time period, we found a significant decline in age-standardised mortality rates in men of on average −2.6% per year from 2004 to 2015 and even of −4.9% per year from 2009 to 2015, where 25- to 84-year-old men had been included (see Table 2). We observed no significant time trend in age-standardised mortality in the total of women aged 25–74 years from 2004 to 2015, whereas from 2009 to 2015, when including up to 84-year-old women, we observed an annual decline in age-standardised mortality rate of −8.0% on average.

**Table 2.** Average Annual Percentage Change (AAPC) in acute myocardial infarction mortality, 28-day case fatality and event rates from 2004 to 2015 by sex and age groups.

	Mortality rate	Event rate	Incidence rate	Recurrence rate	Case fatality
	AAPC (95% CI)	AAPC (95% CI)	AAPC (95% CI)	AAPC (95% CI)	AAPC (95% CI)
Men					
Age groups:					
25–54 years (2004–2015)	0.6 (−1.9; 3.2)	0.0 (−1.1; 1.2)	−0.9 (−3.8; 2.0)	−1.2 (−5.6; 3.5)	0.6 (−1.5; 2.8)
55–64 years (2004–2015)	−1.8 (−4.1; 0.5)	−1.5 (−3.1; 0.2)	−1.8* (−3.2; −0.4)	−2.7 (−5.9; 0.6)	0.0 (−2.6; 2.7)
65–74 years (2004–2015)	−3.8* (−5.2; −2.3)	−2.6* (−5.1; −0.0)	−2.1* (−3.8; −0.4)	−4.7* (−7.5; −1.8)	−1.5* (−3; −0.2)
75–84 years (2009–2015)	−6.0* (−10.5; −1.3)	−3.2 (−6.6; 0.3)	−3.4* (−5.9; −0.9)	−2.8 (−8.4; 3.2)	−3.0* (−4.5; −1.4)
Total population^a^:					
25–74 years (2004–2015)	−2.6* (−3.8; −1.5)	−2.2* (−2.7; −1.6)	−2.6* (−4.9; −0.2)	−3.4* (−6.0; −0.8)	−0.8 (−2.8; 1.3)
25–84 years (2009–2015)	−4.9* (−8.7; −0.9)	−1.7 (−3.8; 0.4)	−2.2* (−3.1; −1.3)	−1.6 (−6.2; 3.2)	−2.9* (−4.7; −1.1)
Women					
Age groups:					
25–54 years (2004–2015)	8.9 (−5.4; 25.5)	1.3 (−3.5; 6.3)	1.9 (−4.4; 8.7)	−4.8 (−10.6; 1.4)	−0.2 (−5.9; 6.0)
55–64 years (2004–2015)	−0.5 (−4.5; 3.7)	−0.6 (−2.6; 1.4)	−2.3 (−5.1; 0.5)	1.7 (−6.5; 10.6)	−0.2 (−4.1; 3.8)
65–74 years (2004–2015)	−2.1 (−4.2; 0.0)	−2.2* (−3.2; −1.3)	−2.3* (−3.7; −0.8)	−3.3 (−6.9; 0.4)	−1.4 (−1.4; 1.7)
75–84 years (2009–2015)	−10.0* (−12.7; −7.3)	−7.7* (−9.4; −5.9)	−9.1* (−12.6; −5.5)	−4.9* (−6.3; −3.6)	−2.1 (−6.7; 2.8)
Total population^a^ :					
25–74 years (2004–2015)	−1.4 (−3.2; 0.4)	−1.3* (−1.9; −0.7)	−1.5* (−2.8; −0.3)	−3.0* (−5.7; −0.2)	0.1 (−1.6; 1.9)
25–84 years (2009–2015)	−8.0* (−9.9; −6.0)	−5.1* (−7.6; −2.5)	−6.5* (−8.9; −4.1)	−2.1* (−4.0; −0.1)	−2.1* (−4.0; −0.1)

AAPC: Average Annual Percentage Change; CI: Confidence Interval.

*AAPC is significantly different from zero at alpha = 0.05 level.

^a^Age standardised.

Regarding age-group specific mortality rates, we observed a significant average annual decrease in the oldest age group in both men and women and in men 65–74 years of age. We found the trends in mortality rates being non-significant within the other age groups, nevertheless in young women (age group 25–54 years) our data indicate towards a possible increase in AMI mortality rate from 2004 to 2015. The joinpoint analysis revealed one significant joinpoint in the years 2006 (details shown in Supplemental Table 1A). However, due to the small number of fatal events within the youngest age group of women, reliable annual percentage change (APC) estimates could not be given.

### Trends in event rates

**Figure 3.**
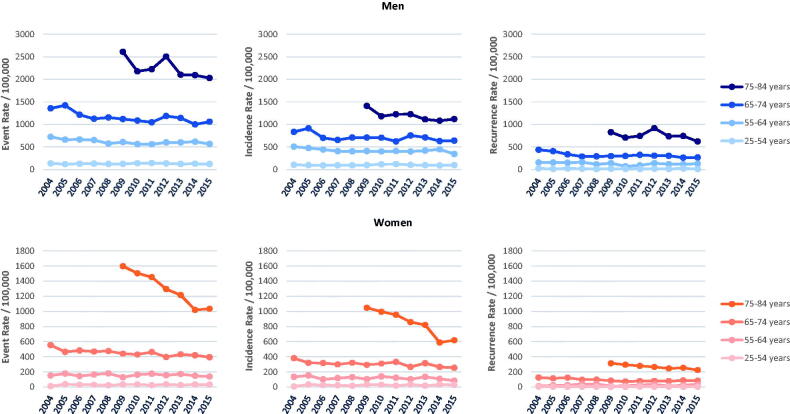
Age group- and sex-specific trends in event rates, incidence rates and recurrence rates of acute myocardial infarction in Augsburg study region, Germany, from 2004 to 2015.

Figure 3 shows higher event, incidence and recurrence rates in men compared to women within all age groups and over the whole investigated time period. In both men and women age group specific event rates were several times higher in the oldest age group compared to those under 74. This difference in event rates was more pronounced in the female population but diminished from 2009 to 2015 due to a decline of event rates in the oldest age group. The proportion of recurrent events in all events increased with age (see Table 1).

As shown in Table 2, age-standardised event rates declined significantly in the total of 25- to 74-year-old men, contributed by declines in both age-standardised incidence rates and age-standardised recurrence rates from 2004 to 2015. When considering the total of 25- to 84-year-old men, the age standardised incidence rate declined significantly, whereas the AAPC in the corresponding age-standardised event and recurrence rate were found negative but non-significant.

When considering age-group specific event rates (Figure 3 and Table 2), in men we found a significant decline in the age group of 65–74 years, contributed by declines in both incidence and recurrence rate. While incidence rates also declined significantly in all other age groups except for the youngest, we observed no further significant time trend in age-group specific recurrence rates in men.

In women, we found significant AAPC decreases in both the total of 25- to 74-year-olds (2004–2015) and of 25- to 84-year-olds (2009–2015) in age-standardised event, incidence and recurrence rates, as summarised in Table 2. The joinpoint analysis showed a higher event decrease in the period from 2011 to 2015 for the total female population aged 25–84 years (APC −6.3, 95% CI −10.9 to −1.5) (see Supplemental Table 1B).

The steepest decline in event rates in women occurred in the oldest age group where the event rate declined by −7.7% per year on average (95% CI −9.4 to −5.9%), contributed by declines in both incidence and recurrence rates from 2009 to 2015 (see Table 2 and Figure 3).

Further, a significant decline in event rate also occurred in 65 to 74-year-old women from 2004 to 2015, contributed mainly by a decline in incidence rate, while we observed a negative but not significant trend in recurrence rate for this age group over the whole time period. Joinpoint analysis revealed a significant decline of recurrence rate in the time period from 2006 to 2010 (see Supplemental Table 1D). Among women up to 64 years we found no statistically significant trends in event, incidence or recurrence rates.

### Trends in case fatality

**Figure 4.**
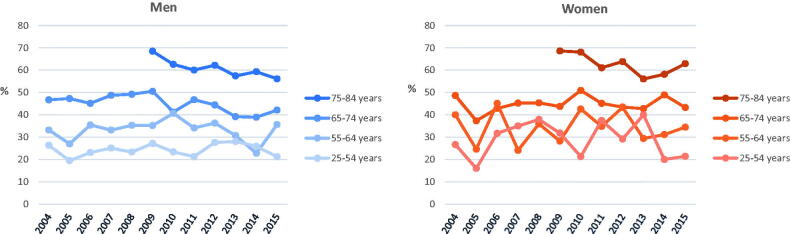
Age group- and sex-specific 28-day case fatality (in percent of all events) of acute myocardial infarction in Augsburg study region, Germany, from 2004 to 2015.

As shown in Figure 4, 28-day case fatality was higher in the oldest age groups compared to the younger in both men and women. Table 2 shows that a statistically significant decline in age-group specific case fatality over the respective time period occurred only in men in the two oldest age-groups (−3.0% and −1.5%).

## Discussion

In the present population-based study, we observed a decline of age-standardised AMI mortality in men aged 25–74 years from 2004 to 2015 of 2.6% per year on average. When considering ages 25–84 years over the time period from 2009 to 2015, age standardised mortality rates declined even steeper and in both sexes.

Age-specific trends revealed that these mortality declines have not been shared equally by all age-groups. In men we observed declines in the two oldest age-groups and in women only in the oldest age group. In women, the drivers behind this mortality decline are presumed to be the observed reductions in incidence and recurrence rate. While for men, mortality rates fell, due to a decline in event rates as well as a decrease of case-fatality.

In young and middle-aged men up to 64 years and in women up to 74 years we found no significant time trends in mortality from 2004 to 2015.

### Comparison with other studies

Our results imply that the overall decline of AMI morbidity and mortality observed previously in the Augsburg study region between 1985 and 2002 [5] continued until 2015. However, the mortality decline reported before had largely been attributable to declines of recurrent cases and case fatality [5,16], while in the present findings from 2004 to 2015, declining incidence rates also contributed to the trend of declining mortality rates. Furthermore, we found the current trends to be inhomogeneous across age groups, especially when including the population up to 85 years of age since 2009, who showed the steepest declines in mortality.

#### Trends in mortality

Our results of age- and sex-specific CHD mortality are in line with other recent studies from within and outside Europe. Steep mortality declines (>4.0% per year) in the population aged 64–84 years on the one hand and smaller or partly non-significant declines among the young population (<55 years) on the other hand had been reported for example from England (2002–2010) [13], the United States (2000–2011) [9] and Australia (1990–2006) [3]. Whereas a recently published registry study from France found a clear decrease in mortality rate in women aged 35–44 [23]. A previous study, analysing German vital statistics from 1980 to 2007, reported declines in mortality rates among under 55-year-old men (between 3.1 and 4.2% annual decline) and women (between 2.3 and 3.2% annual decline) during the last 5–10 years of their study period [6]. We could not find such a decline among this age group, which may indicate the occurrence of a stagnation in mortality rates within the last 12 years.

However, it has to be pointed out that over the whole study period only a small number of fatal events occurred in the youngest age group (women *n* = 132 events, men *n* = 523 events). Therefore, it is challenging to provide statistical evidence concerning mortality trends in this demographic group and the trends we found need to be observed on a longer time scale.

#### Trends in event rates

We found a similar pattern of trends in incidence rates across age groups, which points in the same direction as previous studies from Denmark (1998–2007) [12], Norway (2001–2014) [11], the Netherlands (1998–2007) [14], France (2006–2014) [23], Western Australia (1993–2012 and 1995–2010) [10,15] and Australia (2000–2007) [24].

All of them reported notable declines in incidence rates among middle-aged and older age groups [10–12,14,15]. Several studies reported incidence rates among the younger population (<55 years) declining less steeply compared to the elderly [14], remaining stable [10] or even were reported to have increased in at least one sex [12,15,24].

When considering the event rates, a similar pattern of steeper declines among the older and middle-aged population and comparatively small or absent declines among the younger population, was reported in previous population-based studies from England (2002–2010) [13], France (2006–2014) [23] and from a multinational European study (1985–2010) [1] as well as from Canada (2000–2009) [25], though the latter included only hospitalised events. Considering recurrence rates separately, comparability with current international studies is limited due to different methodologies that have been applied, like only considering hospitalised events and including also milder stages of coronary heart disease [24], not reporting age-specific trends and not reporting hospitalised and prehospital fatal cases combined [26].

#### Trends in case fatality

There were only few comparable international population-based studies overlapping our study period, which reported age-specific case fatality trends including both prehospital deaths and deaths within 28 or 30 days after hospitalisation and included incident as well as recurrent cases [8,13].

Studies from England [13] (2002–2010), Spain [8] (1996/97 versus 2007/08) and France [23] (2006–2014) also found declines of case fatality in men aged 65–84 years, but in contrast to our findings also among younger men (<65 years).

The results differed from ours even more when considering the female population. While we found no significant trends of case fatality in any age-group of women, Smolina et al. [13] reported significant declines among the female age groups ≥ 30 years, Vázquez-Oliva et al. [8] among 35- to 64-year-old women and Meirhaeghe et al. [23] among 35- to 44-year-old women. On the contrary, Dégano et al. [1] who reported aggregated trends from six European nations, even reported an increase in 28-day case fatality in women 65–74 years of age between 2005 and 2010.

### Potential underlying mechanisms of trends

Improvements in prevention, recognition and treatment of modifiable cardiovascular risk factors like smoking, arterial hypertension, hyperlipidaemia and diabetes mellitus might explain declining incidence rates [27]. Of course, due to a higher prevalence of these risk factors in the elderly population, progress in prevention may have had a particularly strong impact on AMI event rates in the oldest age groups [23].

Progress in diagnosing early CHD stage and increased use of evidence-based cardioprotective medication prior to an incident AMI, combined with interventional reperfusion therapy in early stages of ischaemic heart disease might have also contributed to declining incidence rates, at least postponing an incident AMI event to higher age [7,16,28,29].

However, why may the incidence rates have stagnated in the population under 55 years within the last years?

Some recent developments concerning cardiovascular risk factors among the young population in Germany might have counteracted declines in AMI incidence rates. Despite the improvements concerning arterial hypertension in the general population, a rising prevalence and an unsatisfactory level of awareness of arterial hypertension has been noticed among young men (18–29 years old) in Germany [30]. Moreover, the level of unknown and untreated dyslipidaemia remains still high in Germany, especially in the young population [31]. Further, an increase in the prevalence of obesity among young German adults might also have counteracted declining incidence rates [32].

Apart from this, some non-traditional social and psychological risk factors such as depression and perceived stress, have lately been assumed to cause an increased risk of CHD hospitalisations and mortality among young adults [9,33,34]. Hereditary factors are known to play a major role in the pathogenesis of AMI among young adults, which might lead to a smaller possible effect of risk factor reduction on incidence rates for young age groups [11].

Declining recurrence rates and 28-day case fatality might reflect an increased application of evidence-based cardiovascular drug combinations in AMI therapy [35,36] and an increased use of interventional therapy in acute care of AMI patients, which has explicitly been recommended for elderly patients in ACS guidelines since 2000 [36,37].

Given that the oldest age group has the highest case fatality and the largest number of fatal AMI events, this age groups might have benefitted most from improvements in AMI prevention and therapy, being reflected by the steep mortality declines we found among this group.

Since our calculation of 28-day case fatality includes both prehospital deaths and deaths within 28 days after hospitalisation, trends are also influenced by parameters occurring before admission to hospital, for example the timely recognition of coronary symptoms by the patients and health care professionals and the quality of care in the chain of survival before admission to hospital. In women, we found no significant age-specific declines in 28-day case fatality. In any age group. It remains unclear, whether this reflects smaller progress in the treatment of AMI in women or if counteracting trends in prehospital case fatality mask possible improvements in the quality of care after admission to hospital.

### Strengths and limitations

A major strength of our study is the setting in a population-based registry in a defined study region including urban and rural areas and with clinically confirmed AMI cases and validated CHD deaths inside and outside a hospital.

The inclusion of prehospital deaths provides a complete picture of AMI mortality, events rates and case fatality compared to merely hospital-based studies. The standardised verification of clinical AMI diagnoses and data collection by qualified registry staff in each event, provides consistently sufficient data quality. The verification of CHD prehospital deaths and the gain of supplementary details *via* questionnaires to the last treating physician, allows a more accurate assessment of patient characteristics compared to studies solely based on death certificates.

Our study represents the most up-to-date analysis on AMI mortality, event rates and case fatality trends over 12 years in an epidemiological registry-based setting in Germany. Furthermore, we were able to report current trends from the population aged 75–84 years for the first time, which accounts for a high proportion of AMI patients and which has so far not been included in trend analysis from the KORA Augsburg MI registry. However, we were only able to report trends from the seven year-period from 2009 to 2015 for this age group.

A further strength is the consistent use of the same AMI definition criteria for registering cases during the entire study period, since troponin-testing had already been well-established in Germany at the beginning of our study in 2004.

We report trends in different epidemiological parameters contributing to AMI mortality and stratified them by age-groups and sex. Thereby our study provides a detailed overview on the complex pattern of AMI mortality trends and enables to detect disparities in trends across sex and age-groups within the last 12 years.

By nature, information on patients dying outside the hospital or shortly after hospitalisation are limited and the risk of misclassification of an event (or underestimation of risk factor presence) in this group is given, leading to a possible over- or underestimation of event numbers and rates. But as it is unlikely that this has changed over the study period it is unlikely to have resulted in biased trend estimates.

Furthermore, to calculate recurrence rates, we used the mid-year population of the respective calendar year in the denominator and not the population at risk, defined as patients who have survived at least one MI event. Therefore, it is not possible to distinguish whether trends in recurrence rates stem from changes within the numerator (for example less recurrent events due to better secondary prevention) or within the denominator (less people at risk of a recurrent event due to lower incident events).

A further limitation is that our findings are not transferable offhand to other wealthy countries and all-over Germany.

## Conclusion

AMI mortality rates declined from 2004 to 2015 due to declines in event rates and case fatality in the population living in the Augsburg study region. These trends were inconsistent across different age groups with markable declines among the elderly population, indicating that they have benefitted from developments in prevention measures and AMI treatment in recent years. On the other hand, trends among the young population showed alarming signs of stagnation or even increase of AMI mortality rates. These findings emphasise an urgent need for further investigation to verify these trends with focus on the young population and to identify underlying factors leading to it, in order to promptly create better and more effective primary prevention strategies to counteract a possible growing burden of CHD in the future.

Additional Supporting Information may be found in the Supplemental Tables 1(A–E).

## Authors’ contributions

CK, UA and CM conceived the study. CK performed the statistical analyses and drafted the manuscript. UA, CM, IK, MH, BK, CT and JL contributed to data acquisition and interpretation of data. UA, CM, IK, MH, BK, CT and JL critically revised the manuscript for important intellectual content. All authors approved the final version of the manuscript and agreed to be accountable for all aspects of the work.

## Supplementary Material

Supplemental Material

## Data Availability

The data will not be shared. Due to restrictions from Helmholtz Zentrum München, data are available upon request for any researcher based on a standard agreement on data provision within the KORA Research Platform.
